# Remodeling of cardiac passive electrical properties and susceptibility to ventricular and atrial arrhythmias

**DOI:** 10.3389/fphys.2014.00424

**Published:** 2014-11-03

**Authors:** Stefan Dhein, Thomas Seidel, Aida Salameh, Joanna Jozwiak, Anja Hagen, Martin Kostelka, Gerd Hindricks, Friedrich-Wilhelm Mohr

**Affiliations:** ^1^Clinic for Cardiac Surgery, Heart Center Leipzig, University LeipzigLeipzig, Germany; ^2^Nora Eccles Harrison Cardiovascular Research and Training Institute, University of UtahSalt Lake City, UT, USA; ^3^Clinic for Pediatric Cardiology, Heart Center Leipzig, University LeipzigLeipzig, Germany; ^4^Clinic for Cardiology, Heart Center Leipzig, University LeipzigLeipzig, Germany; ^5^Hospital for Children and Adolescents, University of LeipzigLeipzig, Germany

**Keywords:** electrical propagation, passive electrical properties, cardiac tissue, gap junction, connexin, anisotropy, inhomogeneity, cable theory

## Abstract

Coordinated electrical activation of the heart is essential for the maintenance of a regular cardiac rhythm and effective contractions. Action potentials spread from one cell to the next via gap junction channels. Because of the elongated shape of cardiomyocytes, longitudinal resistivity is lower than transverse resistivity causing electrical anisotropy. Moreover, non-uniformity is created by clustering of gap junction channels at cell poles and by non-excitable structures such as collagenous strands, vessels or fibroblasts. Structural changes in cardiac disease often affect passive electrical properties by increasing non-uniformity and altering anisotropy. This disturbs normal electrical impulse propagation and is, consequently, a substrate for arrhythmia. However, to investigate how these structural changes lead to arrhythmias remains a challenge. One important mechanism, which may both cause and prevent arrhythmia, is the mismatch between current sources and sinks. Propagation of the electrical impulse requires a sufficient source of depolarizing current. In the case of a mismatch, the activated tissue (source) is not able to deliver enough depolarizing current to trigger an action potential in the non-activated tissue (sink). This eventually leads to conduction block. It has been suggested that in this situation a balanced geometrical distribution of gap junctions and reduced gap junction conductance may allow successful propagation. In contrast, source-sink mismatch can prevent spontaneous arrhythmogenic activity in a small number of cells from spreading over the ventricle, especially if gap junction conductance is enhanced. Beside gap junctions, cell geometry and non-cellular structures strongly modulate arrhythmogenic mechanisms. The present review elucidates these and other implications of passive electrical properties for cardiac rhythm and arrhythmogenesis.

## Introduction

Although the heart can be considered as a simple mechanical pump, this pump is a highly complex system involving mechanical, electrical, active, passive, and endocrine factors. Many of these factors are subject to remodeling processes in cardiac disease and, thus, are not necessarily constant. This review focuses on remodeling of the passive electrical properties of the heart and its importance for arrhythmogenesis.

Passive electrical properties of cardiac tissue comprise the specific resistance of intracellular and extracellular spaces as well as membrane capacitance. These are strongly influenced by tissue structure. This involves size, shape and arrangement of cardiac cells, including myocytes and non-myocytes, as well as connective tissue, extracellular and intracellular volume conductors and gap junctions. Moreover, anisotropy and inhomogeneities in the spatial (or in some cases temporal) distribution of these factors are of importance.

Electrical anisotropy in the heart refers to differing specific resistances in longitudinal and transverse fiber direction. The elongated shape of myocytes and intercellular coupling mainly at cell poles result in lower longitudinal than transverse resistance. This applies both to the extra- and intracellular space. Spatial inhomogeneities in cardiac tissue, e.g., zones with enhanced deposition of collagen, (fibrosis, which increases with age), lead to a spatial variation of these resistances, which is referred to as non-uniformity. In consequence, cardiac tissue can be considered as non-uniform and anisotropic.

**Figure 1:**
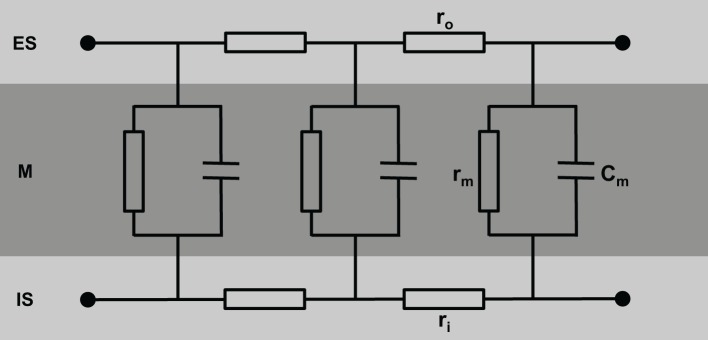
**Schematic view of cardiac tissue modeled as a simple cable consisting of intracellular (r_i_) and extracellular (r_o_) resistors and capacitors (C_m_)**. ES, extracellular space; M, cell membrane; IS, intracellular space.

A simple model of the electrical equivalent circuit of cardiac tissue is a cable formed by cells coupled in series via Ohmic resistors. Each cell in this cable represents a resistor with a parallel capacitor (for review see Weidmann, 1990). The change in voltage is a function of distance (x) according to V_x_ = V_0_ exp(−x/λ) with the length constant λ = √(r_m_/r_i_). The membrane resistance, r_m_, is expressed in Ωcm, the intracellular longitudinal resistance, r_i_, in Ω/cm. The input resistance at *x* = 0 can be described as r_input_ = V_0_/I = r_i_λ. Due to the fiber geometry with radius a, the specific membrane resistance R_m_ equals 2 π ar_m_ [Ωcm^2^] and specific intracellular resistance R_i_ = πa^2^r_i_ [Ωcm]. The specific membrane capacitance can be described as C_m_ = τ/R_m_ with the time constant τ. In a multicellular preparation with parallel running fibers the longitudinal resistance of the extracellular space r_o_ also has to be considered. For these conditions λ is reflected by λ = √(r_m_/(r_i_ + r_o_)) and the conduction velocity θ depends on θ = √(1/(τ_foot_C_m_(r_i_ + r_o_)). This cable theory was originally formulated for nerve axons (Hodgkin and Rushton, 1946) and later on for Purkinje fibers (Weidmann, 1952). It holds true for a continuous cable (Figure 1).

**Figure 2:**
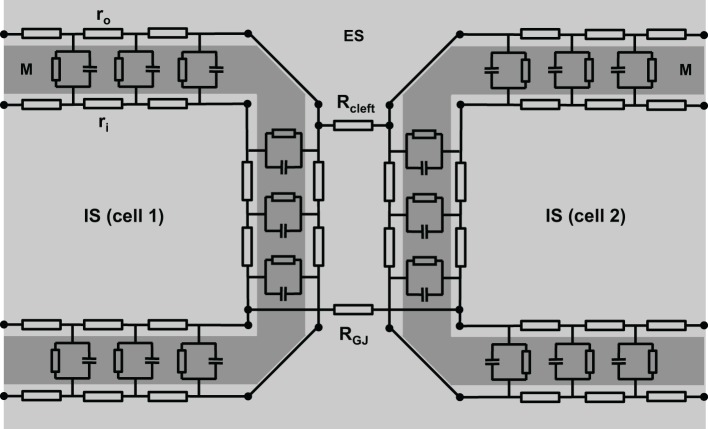
**A more realistic scheme of coupled cardiac cells considering discontinuous properties**. The cell membrane (M) is represented by a series of resistor-capacitor circuits, connecting the extracellular space (ES) with the intracellular space (IS). They are interconnected within one cell via extracellular (r_o_) and intracellular (r_i_) resistors. Gap junction resistance (R_GJ_) connects the intracellular spaces of adjacent cells, while extracellular coupling is realized via the resistance of the extracellular cleft (R_cleft_).

However, this is oversimplifying, since the intracellular space of adjacent cells is connected via gap junction channels. A cluster of single gap junction channels forms a gap junction, which connects the cytoplasm of two adjacent cells by the resistance R_GJ_ (see Figure 2). The gap junction resistance is higher than the resistance of the cytoplasm. Furthermore, the resistance r_o_ of the extracellular space is not homogeneous. The resistance R_cleft_ of the extracellular cleft between two cells near intercalated disks (2–5 nm wide) can be assumed to differ significantly from the much wider clefts elsewhere (>20 nm) not only because of its small width, but also because it contains anchoring proteins and gap junction channels. Therefore, the cable necessarily becomes discontinuous (Figure 2).

Fast sodium channels are essential for impulse propagation. Opening of these channels at the beginning of an action potential generates a depolarizing current (I_Na_), which is responsible for the fast voltage upstroke. Therefore, I_Na_ plays a key role in the propagation of action potentials from cell to cell. It has been shown that these sodium channels are clustered at cell-cell contact zones (Kucera et al., 2002; Maier et al., 2004). This further complicates a correct description of the electrophysiological behavior at cell poles. It also shows that modeling cardiac tissue as a continuum is only reasonable on a macroscopic scale.

Although the equivalent circuit of a discontinuous cable depicted in Figure 2 is more complex than the simple cable, it remains an oversimplification because it does not reflect the geometrical properties of cells and tissue. Cardiomyocytes are typically not shaped like bricks or regular cylinders—as often assumed in mathematical tissue models—but, instead, are of irregular shape with branches interdigitating at the cell poles (Spach et al., 2000). Additionally, cell size shows some variation.

In this regard, it is important to consider the ratio between the cell surface A_m_ and the gap junction conductance g_GJ_ (Seidel et al., 2010): increased diameter of the cell can enhance longitudinal propagation velocity θ_L_ if g_GJ_ is enhanced proportionally to A_m_. If g_GJ_ remains constant, however, increased diameter reduces θ_L_. This becomes clear when considering that A_m_ is linearly related to the cell capacitance C_m_. Thus, it is important to analyze the ratio of g_GJ_/A_m_ in order to understand whether a change in cell size will enhance or reduce θ_L_. Furthermore, transverse g_GJ_ may contribute to longitudinal propagation, which cannot be described by a one-dimensional model (Seidel et al., 2010).

Next, we need to consider the three-dimensional nature of the myocardium and the uneven distribution of fibrotic material, mostly collagen fibers, and fibroblasts. Initially, fibroblasts have been considered electrically silent, but research in the past two decades indicates that electrical propagation seems possible. It has been suggested that myofibroblasts can slow conduction (Rohr, 2004, 2012). Furthermore, it has been shown experimentally that impulse propagation along fibroblast inserts was successful over distances up to 300 μm (Gaudesius et al., 2003). However, it needs to be noted that these cells—although not capable of producing action potentials—were coupled via gap junction channels (see below), while HeLa cells without gap junctions did not enable propagation. Thus, it seems that communication-deficient zones will cause propagation failure.

## How are cardiac cells coupled?

Since the discovery of cardiac gap junctions, which form intercellular channels connecting the cytoplasm of adjacent myocytes, it is established that they represent a low-resistance pathway for electrical propagation (Page and Shibata, 1981). However, gap junction resistance is still higher than the cytoplasmic resistance. Accordingly, impulse propagation along the membrane of one cell is faster than over the intercellular gap junction. The time delay at gap junctions is about 0.21–0.27 ms, and ~0.05–0.1 ms at the cell membrane (Fast and Kléber, 1993; Hubbard et al., 2007).

### Coupling without gap junctions

If one considers neighbored cells as closely packed capacitors, ephaptic coupling via electrical fields might be possible (Sperelakis, 1979). Electrical field coupling (Sperelakis and Mann, 1977) or ephaptic coupling (Copene and Keener, 2008) refers to the initiation of an action potential in a non-activated downstream cell by the electrical field caused by an activated upstream cell. This kind of impulse propagation does not require cytoplasmic connections via gap junctions. It might occur at the intercalated disks, where the membranes of adjacent cells are only 2–5 nm apart.

Accumulation of K^+^ in the junctional extracellular cleft seems necessary to allow sodium channels at the intercalated disk of the upstream cell to fire some microseconds earlier than the channels in the remaining surface membrane. In a theoretical model, this was necessary for effective coupling (Sperelakis and Mann, 1977). However, at present it is unclear whether ephaptic coupling contributes to action potential propagation in normal tissue. Computer simulation studies indicate that, under certain conditions, ephaptic coupling may play a role, but strongly depends on parameters like sodium channel conductance and distribution, and the width of the extracellular cleft at the intercalated disk (Mori et al., 2008; Lin and Keener, 2010). Recent data show that in the perinexus Cx43 gap junctions interact with Na(v)1.5 channels (Rhett et al., 2012). The perinexus has therefore been suggested as the anatomical correlate for ephaptic coupling, the ephapse (Rhett et al., 2013; Veeraraghavan et al., 2014). Local accumulation and shifts of ions may alter local membrane potential. However, there is a lack of experimental evidence, because these local changes on a nanometer scale are difficult to measure. Hence, it remains unclear whether ephaptic coupling significantly contributes to cardiac impulse propagation, especially when considering that, according to the Guoy-Chapman theory, the electrical field near a charged membrane falls to zero within 2–3 nm (Carnie and McLaughlin, 1983).

Experimental evidence suggests that cells need to be coupled by gap junctions to allow the propagation of an electrical impulse: Weingart and Maurer (1988) showed that after manipulating two cells into intimate side-to-side contact, initially there was no transmission of electrotonic potentials or action potentials from one cell to the other. Action potential transfer became possible only after the cells had established new gap junctions. This experiment seems to rule out non-gap junctional mechanisms of intercellular action potential spreading. However, recent evidence was presented for another mechanism of electrical cell-to-cell coupling. Membrane-tunneling nanotubues were suggested as cytosolic bridges between cells (He et al., 2011). This theory, however, still needs further investigation.

### Connexins and gap junctions

**Figure 3:**
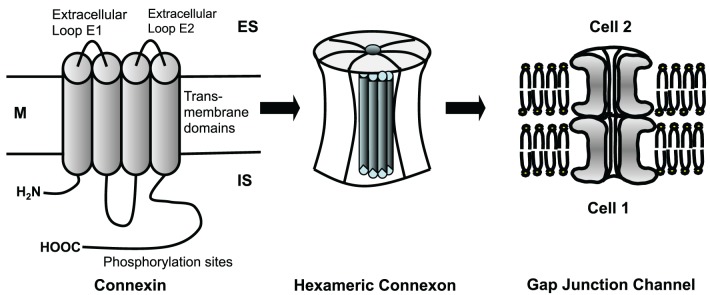
**Connexins are four-transmembrane proteins with two extracellular loops and one intracellular loop**. They build hexameric connexons (hemichannels), which dock to a connexon of an adjacent cell forming a complete, dodecameric gap junction channel. M, cell membrane; ES, extracellular space; IS, intracellular space.

Cardiomyocytes and fibroblasts are homocellularly interconnected by gap junction channels (for review see Dhein, 1998, 2004). Heterocellular coupling between cardiomyocytes and fibroblasts, however, has only been shown in cell culture (Goshima and Tonomura, 1969; Gaudesius et al., 2003) or in the sinus node (Camelliti et al., 2004), but not in living ventricular myocardium. Cells can communicate via these gap junction channels. Each channel is about 150 Å long. The membranes of the two cells are separated in this area by a gap of nearly 20 Å, which is spanned by the channel subunits (Beyer et al., 1995). Cardiomyocytes are connected by intercalated disks, which contain three main structures: the fascia adherens, the desmosome (sometimes called macula adherens), and the nexus. While the fascia adherens is composed of two lipid bilayers separated by 200–300 Å, the desmosome is an almost laminated structure formed by the two membranes. At the nexus both cells are in most intimate contact. This zone contains the gap junction channels, which typically are clustered (Gourdie et al., 1990). A gap junction channel comprises of two hemichannels, which are provided by either cell. Each hemichannel is composed of 6 protein subunits, the connexins. Thus, a gap junction channel is a dodecameric channel with a pore in the center (Figure 3). Connexins belong to a large protein family with at least 21 isoforms in humans. The connexin protein is a four-transmembrane domain protein with an intracellular N- and C-terminal, 2 extracellular loops, and one intracellular loop. For details see Makowski et al. (1977); Chen et al. (1989); Perkins et al. (1997). The most variant part is the C-terminus, which also serves as binding partner for a number of protein kinases, which can control connexin trafficking, assembly, dis-assembly and single channel conductance by phosphorylation. Connexins are characterized by their molecular weight. For instance, Cx43 refers to the connexin with a molecular weight of 43 kDa. The family of connexins is divided with regard to the amino acid sequences and C-terminal length into five subgroups, i.e., α, β, γ, δ, and ε. The genetic names are composed of a “GJ” followed by the family and a number. Thus, Gjα1 means Cx43 which was the first connexin being characterized from the α-group (see http://www.genenames.org/genefamily/gj.php for reference). For a detailed overview on connexins (see Nielsen et al., 2012).

In the heart the predominant connexin isoform is Cx43, which is found in ventricles and atria. Cx40 is mainly found in atrium and in the specific conduction system. Cx45 is mainly found in early developmental stages, in the conduction system, and between fibroblasts and cardiomyocytes. Connexins are synthesized in the rough endoplasmatic reticulum, folded and transported to the trans Golgi network, where they are oligomerized to hexameric hemichannels (connexons). These hexameres are then transported to the plasma membrane and inserted. It is assumed that they flow with lipid rafts within the membrane and accumulate in areas where N-cadherin and zonula occludens-1 protein (ZO-1) are present. At these sites they dock to other hexameres of the neighboring cell forming the gap junction channel. If the connexins are poly-phosphorylated at certain amino acids of the C-terminal, they can be ubiquitinylated and then degraded. Besides the classical proteosomal degradation lysosomal degradation also has been described.

Regulation of gap junction coupling can be realized by modulating the number of channels, i.e., by influencing synthesis, trafficking docking or degradation, or by changes of the single channel conductance. For a detailed review, see Dhein (2004); Salameh and Dhein (2005); Axelsen et al. (2013). Channel conductance depends on the connexin isoform, the phosphorylation state of the connexin, and on the connexin composition of the channel (e.g., homomeric, heteromeric) (Harris, 2001; Moreno, 2004). Interestingly, besides an open and a closed state a single channel can exhibit several conductance states (Harris, 2001; Bukauskas and Verselis, 2004). Moreover, heterotypic channels consisting of hemichannels of different connexin isoforms can show asymmetric voltage-dependent gating. Asymmetric gating refers to the observation that the conductance-to-voltage relationship depends on the polarity of the transjunctional voltage (see e.g., Bukauskas and Verselis, 2004; Schulte et al., 2008).

## Anisotropy and non-uniformity (inhomogeneity)

Anisotropy is defined as the property of being directionally dependent. With regard to cardiac tissue the term is mostly used to describe that specific longitudinal resistivity is lower than transverse resistivity. However, anisotropy values for extracellular and intracellular resistivity differ significantly. Further properties to be taken into account are discontinuity, which means that the fibers are separated by intercalated disks (in contrast to a continuous cable), and non-uniformity, which describes the spatial variation of anisotropy. The latter is due to variations in cellular morphology, cell types (myocytes and non-myocytes) and fibrotic material (collagen) or other non-conducting structures like connective tissue, fat, vessels etc. The difference between uniform and non-uniform anisotropy has many consequences for the pathophysiology of arrhythmia (Spach and Dolber, 1990).

Intracellular resistivity is higher in transverse than in longitudinal direction. Thus, anisotropy in the intracellular space ranges from ~5 to 10 (Clerc, 1976; Roberts and Scher, 1982; Stinstra et al., 2005) and mainly results from cardiomyocyte shape and cellular distribution of connexins. In many cardiac diseases the expression of connexins (Cx43, Cx40, Cx45) is altered. Enhanced levels of Cx43 have been found in cardiac hypertrophy, while in chronic infarction and severe heart failure Cx43 levels are reduced (Severs, 1994; Kostin et al., 2003; Severs et al., 2006). Alterations of Cx43 and Cx40 have been described in atrial fibrillation (Dupont et al., 2001; Polontchouk et al., 2001; Boldt et al., 2003). Since in atrial fibrillation both increased and decreased Cx43 levels have been found, it was suggested that the absolute level may depend not only on the type of arrhythmia but also on the concomitant cardiac pathology (Dhein et al., 2011). In many cardiac pathologies, e.g., chronic atrial fibrillation, cardiac hypertrophy, heart failure and after myocardial infarction, connexins were no longer restricted to cell poles but also expressed at the lateral cell membrane (Polontchouk et al., 2001; Kostin et al., 2002; Cabo et al., 2006). It remained unclear for years whether these lateral gap junctions are functional. In the case of atrial fibrillation, electrophysiological mapping together with immunohistology showed that the lateralization was accompanied by enhanced transverse conduction velocity θ_T_ (Dhein et al., 2011), suggesting that at least a fraction of these lateral gap junctions is functional.

The concept of electrical anisotropy also applies to resistivity of the extracellular space and, besides influencing impulse propagation, has a strong effect on the distribution of epicardial potentials (Roberts and Scher, 1982; Johnston et al., 2001; Stinstra et al., 2005; Schwab et al., 2013). Experimental and computational studies reported anisotropies in the extracellular space ranging from 1.5 to 3.5 (Clerc, 1976; Roberts et al., 1979; Stinstra et al., 2005; Hand et al., 2009). Schwab et al. (2013) distinguished between the two transverse directions (vertical or parallel to the epicardial surface) suggesting differences in anisotropy and pathological remodeling. Their results also indicate spatially non-uniform anisotropy in ventricular myocardium after infarction.

Evidence is growing that the extracellular space, including fibroblasts, plays an important role in cardiac disease and related electrophysiological changes for review see Pellman et al. (2010); Yue et al. (2011); Weber et al. (2013). This is also reflected in the use of bi-domain or multi-domain models for the simulation of cardiac impulse propagation, taking extracellular conductivity and fibroblasts into account (Jack et al., 1975; Peskoff, 1979; Geselowitz and Miller, 1983; Roberts et al., 2008; Sachse et al., 2009). The group of Veeraraghavan et al. (2012) suggested that the effect of gap junction conductance on impulse propagation is modulated by the fraction of extracellular space. They showed that the percentage of extracellular volume was inversely correlated to conduction velocity. This result is surprising because increased extracellular volume should result in lower extracellular resistivity and, according to cable theory, lead to faster propagation. They found that these effects were especially pronounced in transverse fiber direction. However, to increase extracellular volume they used mannitol, which also reduced cell width. A reduction in cell width, however, can strongly slow transverse velocity (Seidel et al., 2010; Toure and Cabo, 2010). Furthermore, with decreasing velocity the effect of gradual uncoupling (higher gap junction resistance) increases because of a switch from continuous to discontinuous propagation, preferably in transverse fiber direction (Seidel et al., 2010). These results show that effects of intra- and extracellular space on impulse propagation are complex and not easy to predict. It remains challenging to measure extracellular conductance and anisotropy in intact cardiac tissue because due to transmembrane channels the measured current between two electrodes will always contain an intracellular component.

As a result of non-uniformity and anisotropy in cardiac tissue fractionated waveforms develop. These have been interpreted as the reflection of discontinuous propagation where each of the multiple negative peaks represents the activation of a small group of fibers (Spach and Dolber, 1986, 1990). Interestingly, aging leads to a change in the tissue's biophysical properties and is associated with increased non-uniformity. This is due to the deposition of collagen fibers between the cells resulting in predominant uncoupling of side-to-side connections (Spach and Dolber, 1986, 1990; Dhein and Hammerath, 2001).

## Source-sink problem

When an action potential propagates, the action potential upstroke velocity and amplitude are larger in the case of transverse propagation together with a shorter time constant of the foot potential τ_foot_. From a theoretical point of view this could mean that longitudinal propagation is more vulnerable to conduction block because of its lower upstroke velocity and amplitude. The increase in upstroke velocity in transverse propagation results from higher resistivity and reduced space constant in this direction. Thus, less current is lost to the neighboring cells (Delmar et al., 1987).

In order to elicit an action potential in a cell, it must receive a depolarizing current from an adjacent, activated cell. The activated cell acts as current source, while the non-activated cell is a current sink with the voltage difference being the driving force for this current. The current transfer is mainly realized via gap junction channels and, to some extent, via the extracellular space. Whether enough current can be transferred to activate a cell is a complex and geometric problem: if a small source (e.g., a tiny strand of activated cardiomyocytes) meets a large sink (e.g., a large area of non-activated cardiomyocytes) the current will flow radially from the activated site to many non-activated sites. Hence, the source current is distributed to many neighboring cells and in each of these the accumulated charge may be too low to trigger an action potential. This will cause conduction failure (Rohr et al., 1997; Rohr, 2004, 2012; Lee and Pogwizd, 2006). In this situation a paradoxical effect can occur: reducing gap junction conductance may preserve conduction by resolving the source-sink mismatch because less current is lost to adjacent sites (Rohr et al., 1997). This is, e.g., realized in the sinus node, where a small current source (sinus node) meets a large sink (atrium). At the sinus node/atrium border there is only little expression of connexins in interdigitating finger-like zones extending from the sinus node into the atrium (Joyner and van Capelle, 1986; Boyett et al., 2006). This reduces the sink because only a small current can flow to a small number of activation sites. In cable theory, this is represented by a smaller space constant leading to a lower capacitive load. On the other hand, if there is high gap junction coupling, the space constant increases leading to a high capacitive load (current sink) and a source-sink mismatch which can evoke conduction failure.

Shaw and Rudy (1997a,b) attempted to describe these phenomena mathematically by the safety factor (SF) of propagation as SF = Q_c_ + Q_out_/Q_in_ (=charge produced/charge consumed). If coupling is reduced gradually the safety factor first is enhanced as a result of the smaller space constant. However, if very low levels of coupling are reached, SF decreases until SF <1 and conduction failure occurs. This happens when gap junctions are almost closed or absent, because then not enough current can be transferred to an adjacent cell before the activated cell repolarizes. In contrast, if I_Na_ is reduced this will result in a progressive reduction of SF. However, propagation velocity will be reduced in both situations (Shaw and Rudy, 1997a,b).

Situations with source-sink problems generally occur when the curvature of the propagating wave front is high. Accordingly, they may be found at the end of Purkinje fibers, during propagation through small isthmuses, around obstacles, and during spiral wave re-entry. Furthermore, source-sink mismatch may occur at the border between normal cardiac tissue and an ischemic zone, when depolarizing current flows into the ischemic region. Since this region is usually non-excitable, it will act as a current sink and, consequently, reduce conduction velocity.

## Arrhythmogenesis

Many definitions of arrhythmias are based on comparing a pathological with the normal heart rhythm. Bradyarrhythmia is a rhythm slower than normal, while tachyarrhythmia is faster than normal. Bradyarrhythmias are often caused by a block or delay of conduction within the specific conduction system, i.e., between sinus node and atrium, between atrioventricular node and bundle of His or within the bundles. If in these structures conduction is impaired, e.g., by a scar resulting from infarction, this will lead to delayed ventricular activation. However, the situation can be more complex involving source-sink problems. As described above, the propagation of an action potential from Purkinje fibers to the working myocardium represents a tissue expansion, which is generally prone to conduction block (Rohr et al., 1997). Both high sodium channel conductance and relatively low intercellular coupling increase conduction safety under these conditions. However, pathological changes like gap junction lateralization or cellular hypertrophy (increased sink), and reduced sodium channel availability (decreased source) can lead to unidirectional conduction block (Kléber and Rudy, 2004; Seidel et al., 2010). If unidirectional block occurs in a Purkinje fiber, this will slow ventricular activation and lead to an abnormal activation pattern. This is part of the reason why Purkinje fibers are considered key players in initiation and maintenance of ventricular arrhythmias (for review, see e.g., Boyden et al., 2010). Besides, unidirectional block can lead to reentry circles resulting in ventricular tachycardia or fibrillation. Thus, bradyarrhythmia can evolve into tachyarrhythmia.

### Reentrant activation

Reentry happens when the electrical activation follows a circular trajectory and is able to re-activate itself at the point of origin. It is a main cause of tachyarrhythmia. Several mechanisms for reentry are known. One common model is explained as follows: if at a bifurcation of the conducting system (e.g., the bifurcation in the anterior and posterior bundle branch) one branch acts as a unidirectional block, allowing conduction only from the periphery backwards, then activation may propagate along branch 2, thereafter across the ventricular muscle, and finally backwards via branch 1. The unidirectional block in this traditional consideration is thought to be caused by a prolonged refractory period in this area. Another classical example describes reentry as a wave which encircles an electrically inactive obstacle, e.g., a scar. This central zone can also be a permanently refractive area, steadily depolarized by the circulating wave, the “leading circle” (Allessie et al., 1977).

Detachment of the reentry wavefront from the obstacle can lead to spiral wave initiation. In this case, a usually non-stationary rotating source moves along a two- or three-dimensional trajectory. The waves initiated from this source show a typical spiral shape (Cabo et al., 1998). It has been shown that the trajectory of the spiral wave core and whether the wave is stable or not, depends strongly on tissue properties like excitability and non-uniformity (Pertsov et al., 1993).

**Figure 4:**
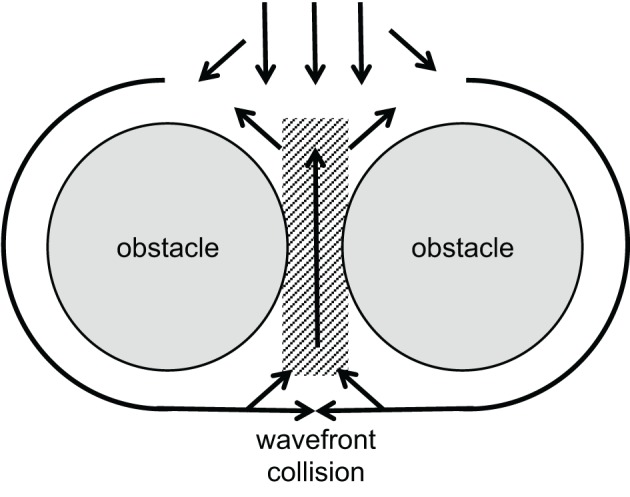
**Schematic illustration of a figure-of-eight reentry**. A wavefront approaches two obstacles separated by a small gap of low excitability (gray area). Conduction is blocked in front of this gap, separating the wavefront. Behind the obstacles, wavefront collision occurs. This increases the current source while reducing the sink and, thus, allows activation of the area between the obstacles. If conduction is slow enough, this leads to reentry in front of the obstacles as shown by el-Sherif et al. (1987).

A certain type of reentrant arrhythmia can originate from a situation in which two closely neighbored obstacles, e.g., collagenous strands or infarct scars, are located transverse to the direction of propagation, with only a small gap of low excitability in between. This structure forces the activation wave to circumvent the obstacles laterally. Behind, the tissue will be activated from both sides causing wavefront collision near the gap. The collision reduces the current sink and increases the source now allowing the activation to propagate through the gap and re-activate the area in front of the obstacles. This kind of reentry has been called “figure-of-eight” reentry mechanisms for ventricular arrhythmias. It may occur in particular when conduction velocity is low (el-Sherif et al., 1987; Lazzara, 1988). See Figure 4 for illustration.

These explanations illustrate very well the observed phenomena, although underlying mechanisms may be a bit more complicated (see below).

### Calcium-induced depolarizations

Under certain pathological conditions like increased adrenergic stimulation or ischemia-reperfusion, cellular calcium overload occurs, leading to spontaneous diastolic calcium waves. These waves reflect spontaneous calcium release from the sarcoplasmic reticulum (Kimura et al., 1984; Marban et al., 1986). The sodium-calcium exchanger then operates in the forward mode generating a depolarizing current. The resulting increase of the membrane potential is often referred to as delayed afterdepolarization (DAD). DADs are considered as potential triggers for arrhythmia because, if big enough, they may initiate an action potential and, subsequently, arrhythmogenic ectopic extrasystoles (Lederer and Tsien, 1976; Xie et al., 2010). However, while it has been shown that a DAD can lead to an action potential in isolated cells (Capogrossi et al., 1987), it remains a matter of debate how a DAD in tissue can overcome the obvious source-sink mismatch (Xie et al., 2010; Tveito et al., 2012). A single cardiomyocyte is, on average, coupled to 4–6 adjacent cells. Under normal conditions, it is therefore impossible that an action potential is initiated. Here, the source-sink mismatch protects from arrhythmogenesis. To overcome the mismatch, there must be a reduced sink via uncoupling or an increased source via DAD synchronization between adjacent cells. Both mechanisms have been suggested to appear under conditions of gap-junctional remodeling (Morley et al., 2005) or adrenergic stimulation (Myles et al., 2012), respectively. Following this argumentation, enhanced coupling should increase the sink and may reduce the risk of arrhythmia. Interestingly, it was shown that the antiarrhythmic peptide AAP10 enhanced gap junction conductance and prevented from certain ventricular arrhythmia (Dhein et al., 1994; Müller et al., 1997; Jozwiak and Dhein, 2008; Hagen et al., 2009). This suggests that increasing gap junction conductance via drug therapy might be an effective strategy for certain forms of arrhythmia. Otherwise, closing of gap junctions could be a mechanism of cardiomyocytes to protect neighboring cells from further damage caused, e.g., by acidosis or calcium overload. It has been shown that gap-junction blockers like heptanol decrease the infarct size after myocardial ischemia (Miura et al., 2004). It is, therefore, surprising that the same effect was shown for gap junction openers like rotigaptide (Haugan et al., 2004), while other studies detected no influence on infarct size (lbye, Nielsen, Petersen, Harlow and Holstein-Rathlou, Xing et al., 2003). Further research is required in this field to better understand these effects and to develop novel anti-arrhythmic drug therapies.

### Effects of hypertrophy

Cardiac hypertrophy is associated with electrophysiological remodeling and an elevated risk of severe arrhythmia (Tomaselli and Marbán, 1999; Kozhevnikov et al., 2002). This is partly due to changes in cellular electrophysiology like remodeling of potassium channels leading to increased action potential duration and elongated QT intervals (Beuckelmann et al., 1993; Swynghedauw et al., 2003). Whether isolated cellular hypertrophy has pro-arrhythmic effects is, therefore, hard to assess experimentally. However, mathematical models have shown that increased cell capacitance increases capacitive load and the discontinuity of action potential propagation. This steepens the relationship between gap-junction conductance and conduction velocity elevating the risk of conduction block both due to uncoupling and source-sink mismatch (Seidel et al., 2010). Furthermore, hypertrophy leads to increased length or diameter of cardiomyocytes, which has both been shown to influence conduction (McIntyre and Fry, 1997; Spach et al., 2000; Seidel et al., 2010). This suggests that hypertrophy itself, in addition to the associated cardiac disease, can be an arrhythmogenic substrate.

### Effects of gap junctions—lateralization and remodeling

Remodeling of the geometrical distribution of gap junctions occurs in a variety of cardiac diseases (Polontchouk et al., 2001; Kostin et al., 2004; Cabo et al., 2006; Salameh et al., 2009). In most cases the fraction of lateral connexins increases. Provided that these connexins form functional channels, they could contribute to arrhythmogenic alterations in impulse propagation (Cabo et al., 2006; Seidel et al., 2010). However, it is discussed controversially since years whether lateral connexins form fully functional gap junction channels. They might as well form hemichannels, which are assumed to be closed in normal physiological situations, or represent a pool of connexins/hemichannels floating in the membrane until they find anchoring site where they form a complete channel with the hemichannel of a neighboring cell. Pathophysiological situations with lateralized gap junctions comprise atrial fibrillation (Polontchouk et al., 2001; Kostin et al., 2002) cardiomyopathy (Kostin et al., 2003, 2004; Severs et al., 2006; Salameh et al., 2009) or myocardial infarction (Cabo et al., 2006). A study investigating atrial tissue from patients suffering from chronic atrial fibrillation found connexin lateralization accompanied by enhanced transverse conduction velocity. Moreover, in the same study metoprolol treatment led to a lower degree of lateralization and lower transverse conduction velocity (Dhein et al., 2011). This provides evidence that at least parts of these connexins form functional lateral gap junctions, although electrode spacing of 1 mm did not allow to precisely link an activation propagation to a certain cell shape. Direct proof needs (a) injection of dyes like Lucifer Yellow into cells within a vital layer of atrial tissue (metabolic coupling), and (b) microscopic mapping of electrical activation with a resolution <10 μm. This should be complemented with connexin immunohistology and electron microscopy of the mapped area and exact superimposition. Theoretical studies have shown potential effects of gap junction lateralization on action potential propagation and possible arrhythmogenic mechanisms. High side-to-side coupling of cardiomyocytes may favor conduction block due to source-sink mismatches (Seidel et al., 2010), while low side-to-side coupling has been suggested to unmask inhomogeneities (Müller and Dhein, 1993; Seidel et al., 2010).

Non-uniformity and anisotropy in cardiac tissue can cause fractionated waveforms. This is increased by a reduction in gap junction protein expression resulting in progression of irregular activation patterns as shown by de Bakker and van Rijen (2006).

### Effects of fibrosis and anisotropic inhomogeneity

Fibrosis is a very common process in cardiac remodeling. When cardiomyocytes are impaired by a pathological event and subsequently undergo apoptosis or necrosis, the remnants are eliminated in the context of an inflammatory process. Finally, the area formerly filled by cardiomyocytes is replaced with connective tissue. This includes fibroblasts and extracellular matrix, mainly collagen. This typical repair process helps to maintain cardiac shape and allows maintenance of intracardiac systolic pressures of about 120–150 mmHg. However, a disadvantage is that this replacement tissue is electrically silent, non-contractile and stiffer than normal myocardium. Fibrotic strands typically are between the cardiomyocytes, running parallel to their longitudinal axis (aligned to the fiber axis), thus separating laterally neighbored cardiomyocytes. Occasionally, there will be a remnant connection between cardiomyocytes, so that the activation pattern follows a zig-zag course with reduced transverse conduction velocity. In situations of a longitudinally propagating wavefront, this type of fibrosis will have only little effect. However, if the wavefronts travels transverse to the longitudinal axis, the fibrotic strand will form an obstacle causing “wavefront curvature” because the traveling wavefront will curve around this insulator (Fast and Kléber, 1997). A similar situation can occur at sudden changes in fiber direction (see “Texture of the Heart”).

The biophysical properties of the fibrotic cardiac tissue can be described as strongly anisotropic (longitudinal resistance << transverse resistance), discontinuous (cells are separated from each other) and non-uniform (local changes in the anisotropic properties) (de Bakker et al., 1993, 1996). The typical ECG under these conditions of highly discontinuous anisotropic tissue exhibits fractionated QRS complexes with reduced amplitude in the case of reduced myocardial mass (Spear et al., 1979; de Bakker et al., 1988).

Fibroblasts, although non-excitable, may allow electrotonic spread of activation, but with considerable delay. Gaudesius et al. (2003) showed in a strand of cardiomyocytes with an interponate of some fibroblasts successful propagation of the action potential, but with a clear delay at the fibroblast site. They showed that the activation can be successfully transmitted over a distance of up to 300 μm filled by fibroblasts. However, it needs to be mentioned that in pathophysiological situations these distances will not be filled with fibroblasts alone but also with a high fraction of collagen. Fibroblasts might couple via gap junctions to cardiomyocytes, which is presumably realized by Cx45 or Cx43, thereby connecting the low-resistance intracellular spaces of fibroblasts and cardiomyocytes. If there is a high fraction of collagen, however, continuous gap junction coupling between myocytes and fibroblasts over long distances becomes very unlikely. Additionally, the ohmic resistance of the extracellular space will be increased. This can lead to conduction failure. It is difficult to estimate the amount of collagenous tissue necessary for electrical isolation, but it will depend on the number of cells in relation to the mass of collagen. Furthermore, it remains unclear whether fibroblast-myocyte coupling occurs *in-vivo* (Kohl and Gourdie, 2014). So far, only one study provides convincing evidence for this possibility in the sinuatrial node (Camelliti et al., 2004), whereas other studies failed to identify heterocellular coupling in the ventricle (Baum et al., 2012).

Different types of fibrosis can be distinguished regarding the texture of collagen deposition: it may be diffuse with small, short, abundant collagenous strands or it may be patchy with larger areas of thick and long collagenous strands. It was shown that the latter type has a higher impact on activation propagation (Kawara et al., 2001). In a computer simulation, discontinuities could be minimized until they vanished when the fibrotic texture was progressively altered from a patchy type to a diffuse type (Pertsov, 1997).

### Texture of the heart

Of note, myocardial fiber direction changes from the endocardium to the epicardium by nearly 90° (Greenbaum et al., 1981). In a normal, non-diseased heart this is a gradual change. However, in diseased hearts, e.g., in cardiomyopathy, myocyte disarray has been observed which leads to zones with a sudden change in fiber direction within a short distance. This can result in a sudden change in resistance since a wavefront traveling along the fiber axis suddenly encounters transverse myocyte strands, so that the electrical resistance for the wave changes. This will cause delay of propagation or wavefront curvatures or discontinuities or unidirectional block (Spach et al., 1982). Interestingly, a similar situation of sudden changes in fiber direction was observed in canine and human pulmonary veins. In this area atrial fibrillation is usually initiated, and this structural particularity may contribute to the arrhythmogeneity of this region (Hocini et al., 2002; Arora et al., 2003).

### Non-excitable regions

Beside myocardial fibers, non-excitable tissue components are present in the heart such as connective tissue (see above) or vessels or fat tissue. These non-excitable obstacles “may cause the formation of self-sustained vortices and uncontrolled high-frequency excitation in normal homogeneous myocardium” (Cabo et al., 1998). In this process the wavefront is thought to detach from the obstacle and form a vortex, i.e., spiral waves are initiated. This process of detachment and shedding of vortices is assumed to depend on wavefront curvature (Cabo et al., 1998).

**Figure 5:**
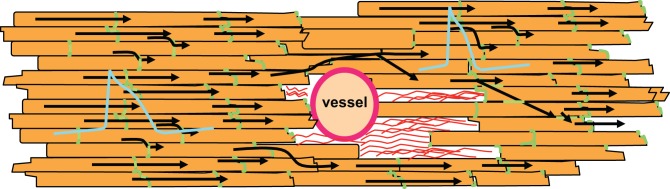
**Non-excitable regions like coronary vessels or connective tissue (shown as red strands around the vessel) lead to changes in action potential duration**. In front of the non-excitable area, current sink decreases leading to longer action potential duration. In contrast, behind this area current sink is increased, which shortens the action potential. Action potential shapes are illustrated in blue, gap junctions in green, cardiomyocytes in orange. Black arrows indicate impulse propagation.

However, there is another characteristic of non-excitable tissue regions: if a wavefront with low curvature propagates, current flows mainly along the potential gradient to the front. Hardly any current is lost to the sides or the area behind the wavefront, because there is no potential gradient. If the wave approaches a non-excitable, electrically insulated obstacle, current flowing to the front will decrease. Thus, in front of an obstacle less current is lost than during normal propagation. As a consequence, the action potential will be prolonged in this area due to the reduced current loss. In contrast, after the wave has surrounded the obstacle it meets unexcited tissue in front and lateral to the wavefront. Thus, current is lost to the sides and the front. As a result, action potential duration will be increased in front and shortened behind the obstacle. It has been shown by epicardial mapping around a coronary vessel that this is indeed the case (Gottwald et al., 1998). For illustration, see Figure 5. Interestingly, this study also showed that in ischemic tissue the difference in action potential duration before and behind an obstacle significantly increases due to slowed conduction. This is a consequence of the ischemic depolarization leading to reduced sodium channel availability, prolonging the time during which current can pass from the excited site to the non-excited sites. This will increase the current loss. Similarly, reduction in sodium channel conductance was shown to enhance local dispersion (variance in action potential duration) at zones of reduced gap junction coupling in a computer simulation (Müller and Dhein, 1993). It seems obvious that, in its extreme form, this phenomenon may lead to unidirectional block, which is a potential trigger of arrhythmia.

### Changes of the biophysical properties by mechanical forces

Mechanical stimuli will lead to local strain of cells, which means an alteration of cell length and length-to-width ratio. In computer simulations it has been shown that changes in cell geometry have an effect on conduction velocity (Spach et al., 2000; Seidel et al., 2010). A local stretch therefore can alter the local pathways of activation (Dhein et al., 2014). On the other hand, it has been shown that structural heterogeneities can affect stretch-induced ventricular arrhythmia (Seo et al., 2010). Wall thickness can be heterogeneously distributed over the ventricle, which will result in a regional modulation of a mechanical stimulus so that regional strain varies. This strain may activate stretch-activated ion channels thereby eliciting cardiac arrhythmia. It was recently shown that local left ventricular stretch leads to local delays in activation so that early and late activation occur closely beside each other (Dhein et al., 2014).

A sudden mechanical insult, an impact or blow by a small spheroid may induce ventricular fibrillation (Kalin et al., 2011). Such an insult is known as commotio cordis. The impact is thought to induce heterogeneity in repolarization within the wall together with a premature depolarization trigger.

## Implications for the clinics (age)

In the daily clinic we also see the importance of the passive electrical properties as, in particular, elderly patients suffer from cardiac arrhythmia. Age has been described to be associated with cardiac fibrosis (Gottwald et al., 1997) and with disturbances of cardiac conduction velocity and enhanced dispersion of action potential duration (Dhein and Hammerath, 2001). According to the latter study, fibrotic strands aligned to the cardiac myocytes result in reduced transverse propagation velocity and thereby enhanced anisotropy. The authors concluded that reduced lateral gap junction coupling may be a typical feature of aged hearts. In accordance with this assumption, they could show that reduction of gap junction coupling in young hearts could mimic the situation found in aged hearts. They also observed that the extracellular potentials were fractionated in areas where activation spread was particularly inhomogeneous. Thus, age-related fibrosis and the resulting disturbances in activation spreading and repolarization indicate that the biophysical passive properties of the tissue are changed and may help to explain why in elderly patients arrhythmia is more frequent than in the young.

Fibrosis is a key characteristic in atrial fibrillation (Boldt et al., 2004), which can be reduced by angiotensin-converting enzyme inhibitors (Boldt et al., 2006). Fibrosis is, among others, considered a major determinant for the development of atrial fibrillation and may lead to structural uncoupling of fiber strands. Moreover, fibrosis enhances the complexity of atrial fibrillation (Verheule et al., 2013). Since according to the present knowledge fibrosis cannot be reversed, this marks a point of no return for atrial fibrillation. Clinical experience underlines that long-standing persistent atrial fibrillation with dilated atria and fibrotic tissue is unlikely to be successfully converted to sinus rhythm for a longer period.

Chronic pump failure leads to remodeling processes in the heart with hypertrophy of the fibers and lateralization of gap junctions. Clinically, it is known that patients suffering from heart failure, cardiomyopathy or cardiac hypertrophy often die from ventricular arrhythmia rather than from pump failure.

Passive electrical properties of cardiac tissue strongly influence myocardial activation, and thereby importantly contribute to the formation of arrhythmogenic substrates.

## Final remark

There are numerous excellent studies on passive electrical properties, gap junctions, arrhythmia, mechano-electrical feedback and related issues. We seriously apologize to those authors who could not be cited.

### Conflict of interest statement

The authors declare that the research was conducted in the absence of any commercial or financial relationships that could be construed as a potential conflict of interest.
